# Ultrastable glasses portray similar behaviour to ordinary glasses at high pressure

**DOI:** 10.1038/srep34296

**Published:** 2016-10-03

**Authors:** C. Rodríguez-Tinoco, M. González-Silveira, M. Barrio, P. Lloveras, J. Ll Tamarit, J.-L. Garden, J. Rodríguez-Viejo

**Affiliations:** 1 Grup de Nanomaterials i Microsistemes, Departament de Física, Universitat Autònoma de Barcelona, 08193 Bellaterra, Spain; 2 Grup de Caracterització de Materials, ETSEIB, Departament de Física, Universitat Politècnica de Catalunya, Diagonal 647, 08028 Barcelona, Spain; 3 CNRS, Inst NEEL, F-38000 Grenoble, France; 4 Univ. Grenoble Alpes, Inst NEEL, F-38000 Grenoble, France

## Abstract

Pressure experiments provide a unique opportunity to unravel new insights into glass-forming liquids by exploring its effect on the dynamics of viscous liquids and on the evolution of the glass transition temperature. Here we compare the pressure dependence of the onset of devitrification, T_on_, between two molecular glasses prepared from the same material but with extremely different ambient-pressure kinetic and thermodynamic stabilities. Our data clearly reveal that, while both glasses exhibit different dT_on_/dP values at low pressures, they evolve towards closer calorimetric devitrification temperature and pressure dependence as pressure increases. We tentatively interpret these results from the different densities of the starting materials at room temperature and pressure. Our data shows that at the probed pressures, the relaxation time of the glass into the supercooled liquid is determined by temperature and pressure similarly to the behaviour of liquids, but using stability-dependent parameters.

Glasses constitute one of the most intriguing materials in condensed-matter^1^. Having a liquid-like disordered structure they behave mechanically as solids. Glasses are typically formed by cooling the liquid at a rate that overcomes the crystallization threat. One of the main features of glasses is the glass transition temperature, T_g_, which characterizes the reversible transformation between the metastable supercooled liquid (SCL) state and the non-equilibrium amorphous solid-like material. The heat capacity of the glass is lower than that of the liquid and, upon heating, the jump in heat capacity marks the onset temperature of devitrification, T_on_. Its value strongly depends on the previous thermal history of the glass and on the heating rate that follows a predefined cooling procedure. Another characteristic feature of glasses is that they age if stored below the glass transition temperature, T_g_, for long periods of time^2^. Aging produces glasses with enhanced stability. The stability of a glass can be established by means of its limiting fictive temperature (T_f_’), i.e. the temperature at which the glass would be in equilibrium with its own liquid. While the glass transition temperature can only be accessed by cooling from the liquid state, the limiting fictive temperature is a property of the glass. The enthalpic T_f_’ is obtained by integration of the normalized heat capacity curve that is measured during a calorimetric cooling scan or during a calorimetric heating scan starting from a given glassy state. Improving the glass stability by aging is a rather inefficient process due to the exponential increase of the relaxation time (or viscosity) below the glass transition temperature. A breakthrough in the field was the recent discovery that vapour-deposition can produce glasses that rival in stability with ambers naturally aged for millions of years^3^. Those glasses, dubbed ultrastable glasses (UG), are typically grown at temperatures around 0.85 T_g_, where T_g_ stands for the conventional glass (CG) transition temperature measured when the liquid is cooled at 10 K/min^4^,^5^,^6^,^7^,^8^. Besides the enhanced kinetic and thermodynamic stability, vapour-deposited stable glasses have been shown to exhibit striking properties with respect to a conventional glass obtained from the liquid. Among them, higher densities and higher sound velocities which imply higher modulus^9^,^10^, surface-initiated transformation mechanism into the supercooled liquid in thin films^11^,^12^,^13^,^14^, absence of TLS (tunnelling two-level systems) in Indomethacin (IMC) at cryogenic temperatures^15^ and lower heat capacities and thermal expansion coefficients^16^. In particular, ultrastable IMC glasses, one of the archetypical UG’s^3,5^, have a higher density by about 1.2% and a lower heat capacity of the glass by about 4%^17^.

While the properties of the glass transition temperature have been deeply studied as a function of temperature by calorimetry in many different glasses, the pressure dependence of the calorimetric glass transition is a subject relatively little explored^18^. The main reason can be attributed to experimental difficulties, in relation to applying high pressures in calorimetric experiments. On the contrary, dielectric or Pressure-Volume-Temperature (PVT) measurements are more abundant and permit to broadly infer several tendencies with respect to molecular interactions^19^,^20^. For instance, it has been found that glasses with strong molecular interaction of hydrogen bonding type, systematically show lower values of dT_g_/dP compared to glasses dominated by van der Waals forces^18^,^21^,^22^,^23^. Another universal feature of glasses is that over a sufficiently large pressure range the pressure dependence of T_g_ is non-linear, i.e. the effect of pressure on temperature weakens when pressure increases and can be adjusted with the empirical Andersson-Andersson equation^24^,


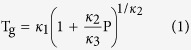


where *κ*_1_, *κ*_2_ and *κ*_3_ are empirical constants. Davies and Jones derived, based on the Ehrenfest equations, two expressions for dT/dP in the liquid state evaluated at T_g_^25^. One of these expressions has been found to describe a large range of materials^23^:


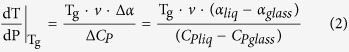


where ∆α and ∆C_p_ refer to the difference in isobaric expansivity and heat capacity at T_g_, between the liquid and its corresponding glass, and *v* is the molar volume at T_g_. We are not aware of previous studies that analyse the pressure dependence of aged glasses over a wide range of stabilities.

In a previous work, we developed an empirical model that could simultaneously describe the relaxation time of the liquid and of glasses of different stability^26^. The model was built with data taken at ambient pressure and therefore only depends on temperature and density. What would be the effect of pressure on glasses of different stability? Can we explain the new data measured as a function of pressure introducing a density dependence on pressure? We present in this work measurements of the devitrification temperature of ultrastable and conventional IMC glasses as a function of pressure. We also propose a tentative extension of our previous empirical model that aims to describe the relaxation dynamics of the system as a function of temperature and pressure by considering the dependence of density on these variables.

## Results

### Evolution of the onset of devitrification as a function of pressure

**Figure 1:**
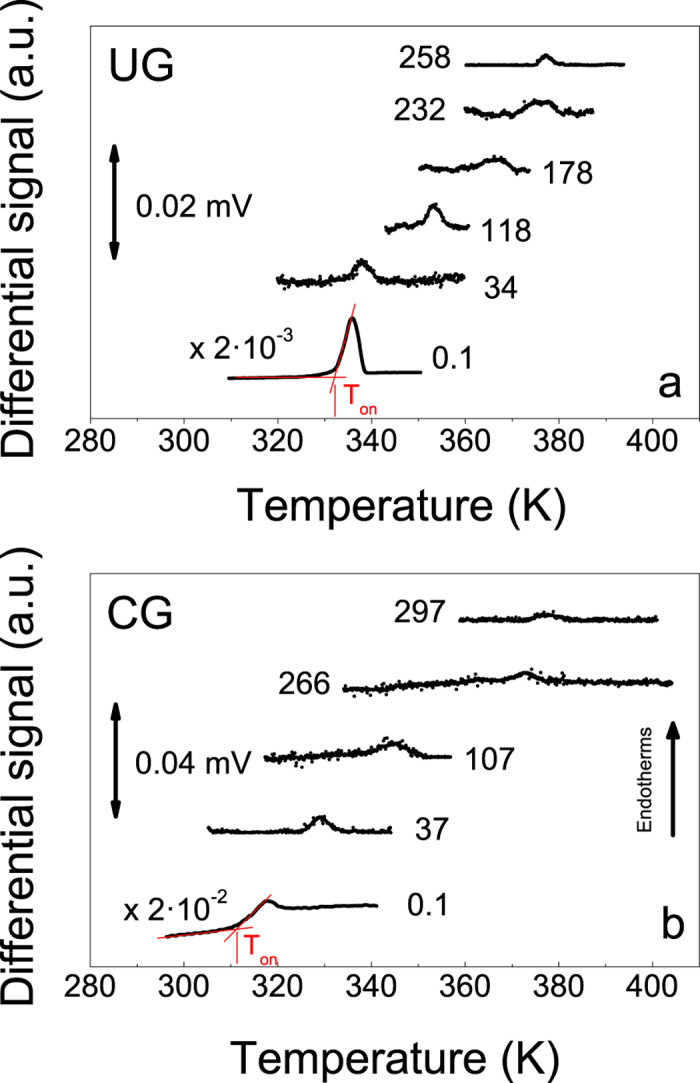
HP-DTA signal as a function of temperature for different pressures, in MPa, as indicated in both figures, measured on heating at 2 K/min. (**a**) Ultrastable IMC glass grown from the vapour phase at a substrate temperature of 0.85T_g_. (**b**) Conventional glass formed by cooling the liquid at 2 K/min. The curves at atmospheric pressure correspond to specific heat measured by DSC. The onset temperature of devitrification is calculated as indicated by the red lines in the figure. The curves have been shifted vertically for clarity.

Two sets of Indomethacin samples, 20–40 μm thick UGs with T_f_’ = 280 K, and CGs obtained by cooling the liquid at 2–10 K/min, with T_f_’ = 315 K, were temperature-scanned at pressures ranging up to 300 MPa in a home-made high-pressure differential thermal analyser (HP-DTA). The values of T_f_’ at ambient pressure were determined from differential scanning calorimetry (DSC) measurements, as detailed in the methods section. The calorimetric curves obtained at different pressures, for both ultrastable and conventional glasses, are shown in Fig. 1. It is apparent for both glasses how the onset of devitrification shifts to higher temperatures as pressure increases.

**Figure 2:**
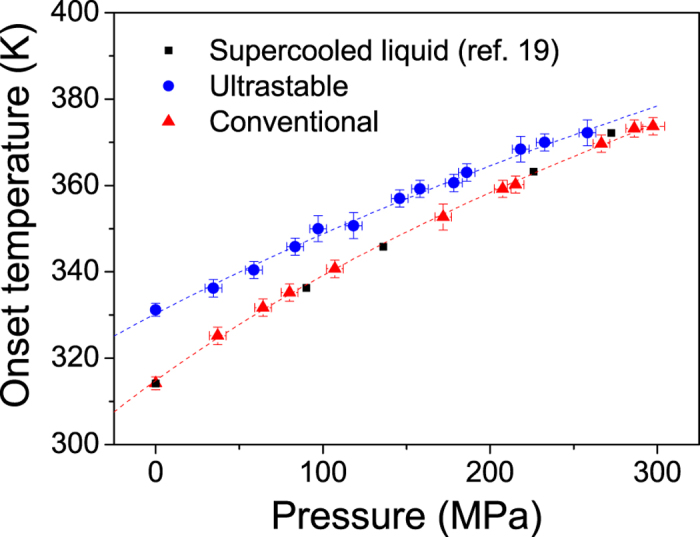
Onset of devitrification temperature versus pressure for IMC ultrastable (blue) and conventional (red) glasses, obtained from the calorimetric data shown in Fig. 1. The experimental data have been fitted using equation (1) (dashed lines). The parameters are κ_1_ = 314.85 K, κ_2_ = 4.68 and κ_3_ = 1124 for the conventional glass and κ_1_ = 330.31 K, κ_2_ = 4.012 and κ_3_ = 1637 for the ultrastable glass. The black dots correspond to data from ref. 19.

Besides possible pressure effects, the shape and the smaller overshoot of the DTA signal at the onset of devitrification, T_on_, for pressures above 0.1 MPa are due to the lack of sensitivity of the HP-DTA setup, precluding a proper evaluation of the limiting fictive temperature as a function of pressure. Therefore, we will concentrate the analysis on the evolution of T_on_ as a function of pressure for both types of glasses (Fig. 2). We also note that onset temperatures obtained for CGs produced by cooling the liquid at normal pressure and then pressurizing the glass before carrying on the temperature scan or, alternatively, obtained by cooling the liquid at high pressure, were the same within the experimental uncertainty of our HP-DTA setup. The observed reversibility between temperature and pressure does not necessarily hold for all glasses. While an increase of pressure in the conventional IMC glass or in the supercooled state induces a similar dynamic response, an increase of pressure in an ultrastable glass may have a different response. Therefore, if one could obtain a highly stable (aged) glass by cooling from the supercooled liquid, a change of pressure in the glassy state and a change of pressure in the liquid state, followed by a decrease of temperature could yield different final glassy systems.

The T_on_ vs. pressure data have been fitted using equation (1) (dashed lines in Fig. 2). For the CG (dT_on_/dP)_Patm_ evaluated at P = 0.1 MPa yields a value of 280 ± 22 K/GPa. The CG was produced and measured at the same cooling/heating rate around 2 K/min. Therefore, the devitrification temperature evaluated on heating, T_on_, and the glass transition measured on cooling, T_g_, coincide, i.e. 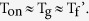 From this we infer that dT_on_/dP = dT_g_/dP. In fact, our experimental value is in relatively good agreement with previous experimental data reported by Wojnarowska *et al*. using dielectric spectroscopy^19^, who obtained 254 K/GPa (black points in Fig. 2). This value suggests that van der Waals interactions dominate over hydrogen bonding, as typically found in polymers and other van der Waals glass-formers^18^,^23^. Furthermore, the experimental value of (dT_g_/dP)_Patm_ for the CG agrees remarkably well (within 3%) with the one calculated from equation (2) using available data from literature for glass and liquid specific volumes, thermal expansion coefficients and heat capacity jump (Table 1). The slope of the UG at P = 0.1 MPa is 201 ± 24 K/GPa, approximately 30% lower than the value obtained for the conventional IMC glass. This may be an indication of the existence of a higher degree of strong intermolecular interactions, such as hydrogen bonds, compared to conventional IMC, a tendency already reported in other works^27^,^28^.

The direct comparison of the experimental data dT_on_/dP for the ultrastable glass with equation (2) can be questioned since the devitrification temperature measured on heating, T_on_, for the UG is different to the glass transition temperature evaluated on cooling. Ultrastable glasses are somehow equivalent to glasses cooled at extremely low cooling rates leading to T_on_ > T_g_ if the glass is reheated at conventional heating rates, i.e. 1–10 K/min. In this sense, the experimental dT_on_/dP (201 K/GPa) and the value of dT_g_/dP calculated using equation (2) (255 K/GPa) are not directly comparable. We could use the value of the limiting fictive temperature as a potential indicator of the validity of equation (2) for the UG, since T_f_’ can be considered very close to the T_g_ of a hypothetical glass obtained by cooling the liquid at the equivalent cooling rate^29^. In fact, using T_f_’ in equation (2), dT/dP_T = Tf’_ yields a value of 210 GPa/K, much closer to the experimental value of dT_on_/dP at T_on_. If we assume that fictive temperatures are affected by pressure in the same way as the glass transition temperature T_g_, then it would be natural to use the generalized limiting fictive temperature, as a trace of the validity of the Ehrenfest-type relationship^30^,^31^. Although we have no theoretical framework supporting the validity of this experimental observation, it seems logical that the Ehrenfest relations can be applied at the point where the thermodynamic parameters of the glass are equal to those of its corresponding equilibrium liquid.

Interestingly, extrapolation of the Andersson-Andersson function (equation (1)) to higher pressures seems to yield a completely different scenario where the onsets of devitrification of both UG and CG, as well as dT_on_/dP, approach each other as pressure increases. The dynamics and the thermodynamic state of vapour-deposited UGs at ambient pressure are clearly different with respect to CGs, cooled from the liquid. The different onset of the calorimetric glass transition temperature and the different value of the limiting fictive temperature at P = 0.1 MPa for UG and CG can be related to the change of the energy barriers between meta-basins and their different energy position in the energy landscape respectively. At ambient pressure the difference in onset of devitrification is 20 K, while at higher pressures it is significantly reduced. i.e., at high pressure both glasses transform into the SCL at a similar temperature. Since during cooling/heating at 10 K/min the relaxation time of the system equals approximately 100 s at the transformation temperature^19^, T_on_(CG) ≈ T_on_(UG) implicitly means that they share a common relaxation time at that temperature. This is a dramatic change, since at ambient pressure the variation in relaxation times between ultrastable and conventional glasses of many glass-formers is 4–5 orders of magnitude^4^,^7^,^13^,^26^.

**Figure 3:**
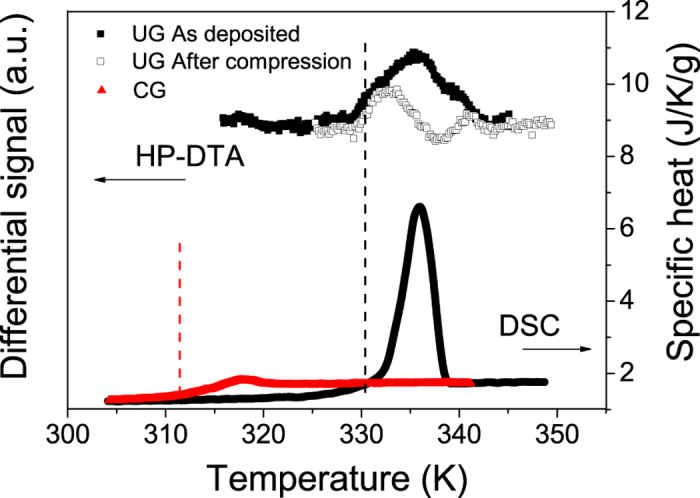
Upper curves: HP-DTA curves, showing the differences between an ultrastable IMC glass submitted to 300 MPa (black open symbols) and another without any pressure treatment (black solid symbols), both of them measured at ambient pressure and at 2 K/min. Differences in the shape of the after-compression signal are due to technical reasons. Lower curves: DSC scans from a conventional IMC glass, cooled from the liquid at 2–10 K/min (red curve) and from an ultrastable glass (black curve), both measured at ambient pressure. Vertical dashed lines indicate the position of the onset of devitrification.

Unfortunately, the experimental setup does not permit an accurate evaluation of the limiting fictive temperature at high pressures and therefore precludes finding a direct relation between T_f_’ and pressure. Most of the prior measurements at high pressure have only access to dynamic properties of the system and the resulting information is not directly connected to the thermodynamics of the glass itself. However, to test whether a pressure change leads to irreversible changes in the structure of the glass we carried out an additional experiment. The methodology consisted on exposing a UG glass to a pressure of 300 MPa at room temperature. We then returned the glass to ambient pressure to subsequently perform a temperature scan. Figure 3 shows the calorimetric curves for a pressurized and an unperturbed glass measured at ambient pressure in the pressure-DTA setup. As has been mentioned before, the sensitivity of this setup is rather limited and the shape and area of the transition peaks are not reproducible. Furthermore, the effects of pressure on the container crucible may yield variations in some features of the transition peak. However, the onset of the transition is indeed accurate and, as can be seen in the figure, both glasses show the same onset. Moreover, when comparing these two curves to measurements performed in a DSC (lower curves) on a UG glass, we can see how the onset of the transition is similar. This is by no means obvious, since according to the values of compressibility reported in the literature^9^, a pressure of 300 MPa should change the volume of the glass by ~4%.

### Glass relaxation time as a function of pressure

It has been shown that van der Waals’ bonded liquids and polymers obey power-law density scaling^32^,^33^,^34^, which means that the average relaxation time of the liquid is a function of Tv^γ^, where v(T, P) = 1/ρ is the specific volume and γ is a material constant. Casalini *et al*.^33^ derived an expression, 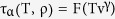, considering that the relaxation time is governed by the entropy of the system, S_c_, as the Adam-Gibbs model proposes, but using a generalised equation for S_c_ that takes into account the influence of both temperature and, also, pressure (or, equivalently, changes in specific volume). In particular,


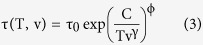


where 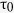 and ϕ are constants and 
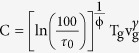
, with T_g_ the conventional value of glass transition temperature for IMC, 315 K, and v_g_ the specific volume of a CG at that temperature. γ is a material-dependent constant, that is generally identified with the Grüneisen parameter, although the exact association is still under discussion^34^.

Although this description is generally applied to equilibrium liquids, we concluded in a previous work that the relaxation times of glasses with different stability, as well as the supercooled liquid, can be described by equation (3) choosing the adequate stability-dependent parameters in the expression for v(T, T_f_’)^26^. The proposed model was applied to data obtained at ambient pressure, as were all the relations used for the parameters of the model, such as the dependence of density on temperature. We suggest that the same model can be applied to the data presented here by introducing in the mentioned equations the dependence of density on pressure.

According to the Murnaghan equation of state^35^, the bulk modulus of a system, K_T_, can be expressed as a linear function of pressure,


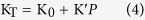


where K_0_ is the bulk modulus at ambient pressure and K′ accounts for the linear variation of K_T_ with pressure. The dependence of K_T_ on temperature is typically small and is considered as a small perturbation at very high temperatures. In the temperature range explored in this work, we impose the bulk modulus to be constant in relation to temperature changes. Integrating the expression for the isothermal bulk modulus, 
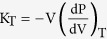
, and using equation 4, the specific volume of a system can be expressed as a function of pressure. The density, inverse of the specific volume, takes the following form,


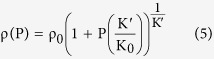


where ρ_0_ is the density at normal pressure. Since the temperature range probed in this work is relatively small, we argue, as it was done when deriving the parameters used in equation (3), that the dependence of density on temperature can be considered linear. Introducing the effect of pressure given by equation (5), we obtain


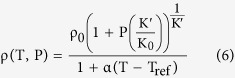


The variation of the thermal expansion coefficient with pressure is related to the variation of the bulk modulus with temperature as 
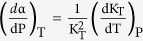
. Since the dependence of K_T_ with temperature is small relative to K_T_, we consider 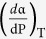 to be negligible. We also note that we have considered no dependence of the thermal expansion coefficient on temperature.

**Figure 4:**
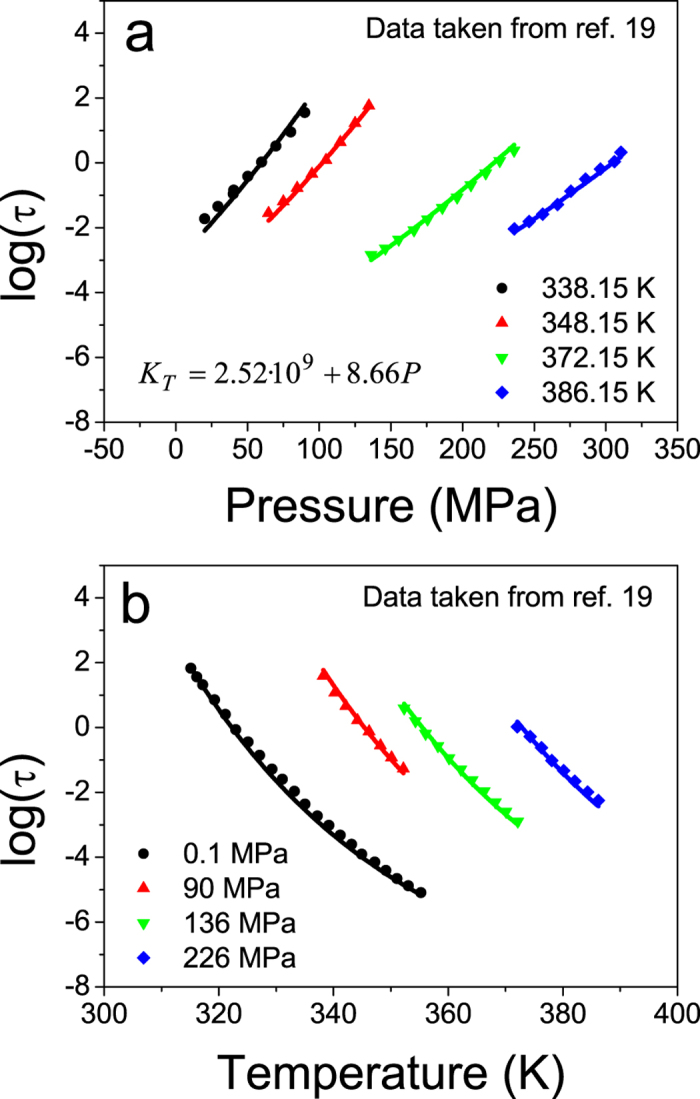
Relaxation time of supercooled IMC liquid extracted from ref. 19 at (**a**) different temperatures and (**b**) different pressures. Data are fitted using equation (3), introducing the dependence of density on pressure described by equation (6). All parameters appearing in equation (3) are taken from ref. 26. Only the bulk modulus has been allowed to adjust, yielding 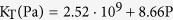.

We show in Fig. 4 reported values from Paluch and co-workers of relaxation time of supercooled IMC liquid measured at different temperatures and pressures^19^. We use these data to infer the values of K_0_ and K′ for the supercooled IMC liquid by fitting the curves using equations (3) and (6), where the values of 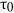, C, ϕ, γ, α and T_ref_ have been extracted from ref. 26. The fit of the data yields K_T_ = 2.52 · 10^9^ + 8.66P for the bulk modulus of IMC supercooled liquid.

We now focus on the relaxation time of glasses. In order to infer the value of K_T_ (equation (4)) for IMC glasses of different stability at ambient pressure, K_0_, we use the adiabatic bulk modulus, K_S_, reported by Kearns *et al*. at ambient pressure^9^. The adiabatic bulk modulus and the isothermal bulk modulus can be related by 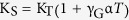
36, where α is the thermal expansion coefficient and γ_G_ is the Grunesein parameter. The Grüneisen parameters for the two glasses can be calculated from thermodynamic quantities^26^, yielding 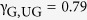 and 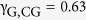. We obtain 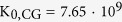 Pa and 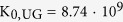 Pa for the bulk modulus of CG and UG at atmospheric pressure, respectively. Further details can be found in the Supplementary Information. We note that the use of a different value of γ_G_ does not alter the conclusions reached below, but only the absolute value of K_T_.

**Figure 5:**
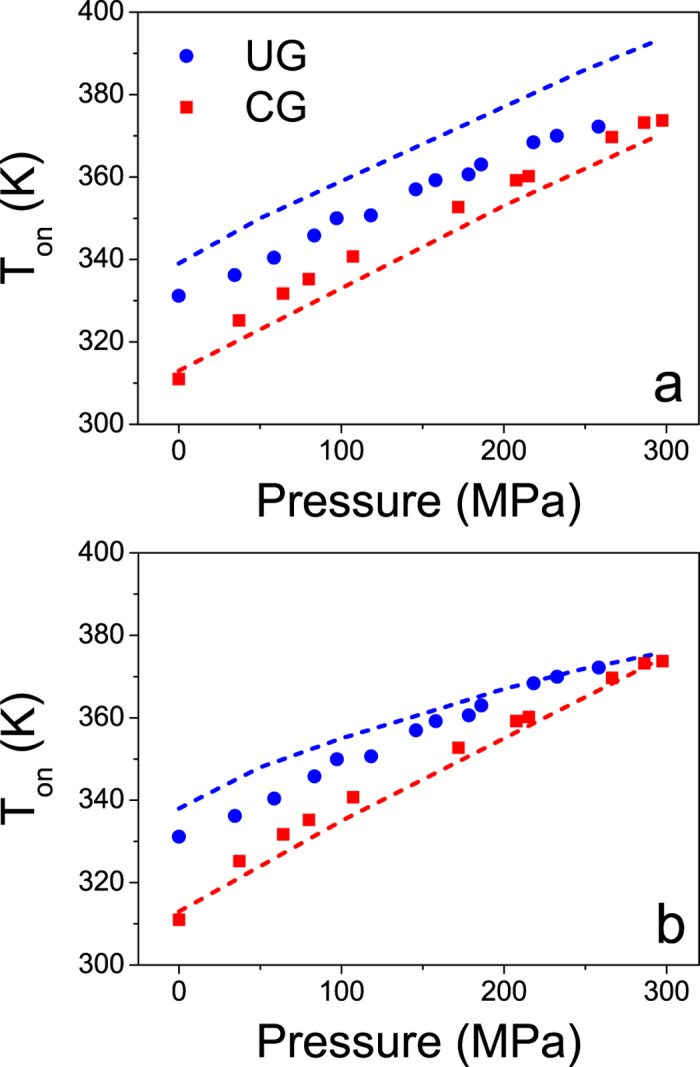
Comparison between experimental data of T_on_ as a function of pressure for UG and CG (solid symbols) and values predicted using equation (3) (dashed lines), taking (**a**) K′ constant (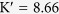) and (**b**) system-dependent value of K′ (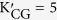, 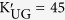). The values of K_0_ used in the two plots are 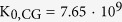 Pa and 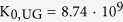 Pa, from the expression 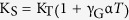.

Since this set of reported data has been measured at ambient pressure, no information regarding K′ can be derived. Therefore, we will consider two alternative plausible scenarios. First, we consider that K′ remains unaltered after vitrification and, therefore, glasses and liquid have the same K′ value, 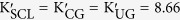. By introducing the dependence of density on pressure in equation (3), and using the parameters found in ref. 26, we can infer the relaxation time of both glasses as a function of temperature and pressure, 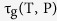. The temperature at which 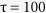  s is considered as the onset of devitrification^19^. We have plotted these temperatures in Fig. 5a for the conventional and ultrastable IMC glass for different values of pressure (dashed lines in Fig. 5). We find that, under the assumption of invariant K′, the onset of devitrification of conventional and ultrastable glasses do not seem to approach at high pressures, contrary to our experimental results. As a second scenario, we assume that: i) the bulk modulus of glass and liquid respond differently to pressure changes, and ii) that this response depends on the stability of the glass. Considering the calculated values of K_0_, we speculate that the value of K′ follows the same tendency, i.e. the larger the thermodynamic stability of the glass the higher the values of K_0_ and K′ are. In particular, we find that we can qualitatively describe the experimental data shown in Fig. 2 by assuming 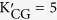 and 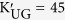 and using equations (3) and (6), as can be seen in Fig. 5b. We note that, according to this model, the density of each glass is different, as well as its dependence with pressure. A similar result was deduced from the application of the mean-field theory on glasses with different stability^37^.

Based on the above description we tentatively propose that the different curvature in Fig. 2 is due to the different bulk modulus between the conventional and ultrastable glass. In particular, ultrastable glasses not only have a higher bulk modulus than CG at ambient pressure, as was already reported^9^, but also this value is more affected by pressure than the bulk modulus of the liquid or conventional glass.

**Figure 6:**
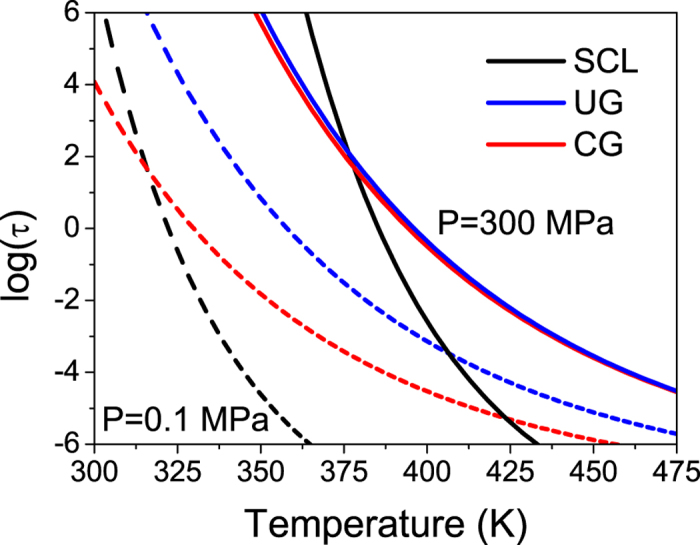
Scheme of the relaxation time of IMC SCL, CG and UG at ambient pressure (dashed lines) and at 300 MPa (solid lines) under the assumptions explained in the text. At 300 MPa, the difference between glasses of very different stability almost vanishes.

Under this framework and considering the relationship between density and relaxation time expressed in equation (3), the experimental data shown in Fig. 2 would depict a scenario in which relaxation dynamics of glasses with very different stabilities at ambient pressure (ΔT_f_’ = 35 K), have similar relaxation dynamics at sufficiently high pressures. A representation of this scenario is given in Fig. 6. In other words, high pressure would make, from our experimental point of view, glasses of different stability practically undistinguishable. We remark that by using equations (3, 4, 5, 6) to describe the dynamics of ultrastable and conventional glasses, we are implicitly assuming that the observed differences between these two systems mainly originate from their distinct density values at atmospheric pressure and to the different density variation with the thermodynamic parameters, pressure and temperature. From this point of view one could infer that an ultrastable glass behaves similarly with pressure as a highly aged glass. Although more data is necessary in order to identify the specific dependence of density with pressure, the analysis developed here permits us to tentatively extend the relaxation time model that described the behaviour of a liquid and its glasses of different stability to include the influence of pressure. The analysis presented in this work, would indicate that the relaxation time of glasses of different stability will converge in the high pressure limit.

## Conclusions

We have analysed the pressure dependence of the glass-to-liquid transformation in two glasses of indomethacin that have extremely different values of limiting fictive temperature, ∆T_f_’ = 30 K. The two glasses show a different dependence of the temperature of devitrification on pressure when evaluated between normal pressure and 300 MPa. This variation could be related to the differences in packing and molecular binding of the two glasses. Interestingly, extrapolation to high pressures, shows that both glasses would share the same onset temperature and the same (dT_on_/dP)_Patm_. Preliminary results show an invariance of the onset temperature of ultrastable glasses before and after submitting the sample to high pressure, an indication that pressure would not irreversibly affect the stability of the glass.

We extend the joint description of relaxation dynamics of glasses and liquids, including a particular pressure dependence of the glass and liquid density through the bulk modulus of the system, K_T_ = K_0_ + K′P, where K_0_ is the isothermal bulk modulus at ambient pressure and 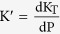. Under this assumption, we find that the measured experimental data can be satisfactorily described considering a system-dependent value of K′, i.e. different glasses and liquids have different values of K′. While further experiments are required to corroborate this assumption, its verification would imply that i) we can extend our relaxation time generalization, at least qualitatively, to variations of pressure by assuming a particular dependence of density on pressure and ii) the bulk modulus of glasses with different stability and the liquid would be differently affected by pressure.

According to the unified description of glass dynamics, the relaxation time of glasses at high pressure converges towards a unique value, in analogy to the effect of temperature on glasses with different stability, that converges to a unique value of relaxation time at high temperatures.

## Methods

### Sample Preparation

Indomethacin (IMC) films with thickness ranging 20–40 μm were grown by thermal evaporation within a UHV setup with base pressure of 5 × 10^−9 ^mbar. The growth rate was fixed to 0.12 ± 0.02 nm/s and the deposition temperature was set to 266 K, 0.85 T_g_, values that produces glasses with high kinetic and thermodynamic stability. The films were grown onto aluminium foil to introduce sufficient mass (100 mg) in the calorimetric vessels and enable subsequent high-pressure experiments. Conventional glasses of IMC were prepared by cooling approximately 200 mg of melted IMC at a cooling rate of around 2 K/min directly into the calorimetric vessel at ambient pressure. Crystalline IMC powder with purity higher than 99.9% was purchased from Sigma-Aldrich and used without further purification.

### Measurement Protocol

The heat capacity of IMC ultrastable and conventional glasses at ambient pressure was measured by Differential Scanning Calorimetry (DSC) with a Perkin Elmer DSC7. To calculate the limiting fictive temperature of each type of glass we have followed the procedure described by Moynihan *et al*.^38^. Basically, the limiting fictive temperature is determined by the intersection temperature between the enthalpy of the liquid and the enthalpy of the glass, which are obtained by integrating the specific heat as a function of the temperature.

Experiments above ambient pressure were carried out with a home-made high-pressure differential thermal analysis (HP-DTA) setup, described in detail elsewhere^39^. Essentially, the temperature of the irimo calorimeter block is surrounded by an external resistance for temperature linear changes controlled by a Pt-100 thermometer embedded in the block. Sample and reference temperature sensors are calibrated K-type (chromel-alumel) thermocouples which are inserted into Sn cylindrical pans. The pressure into the calorimetric block was transmitted by compressing a liquid and measured by calibrated Bourdon gauges with an accuracy better than 0.5 MPa. Because pressure increases concomitantly with the temperature increase during the scan, the error in P_g_ is intrinsically linked to the error in T_on_, which is mainly associated with the onset uncertainty. For the ultrastable glass, the as-deposited samples together with the Al foil substrate were mixed with an inert perfluorinated liquid (Galden^®^ from Bioblock Scientifics, Illkirch, France) before sealing to guarantee the hydrostatic transmission of pressure as well as to ascertain that in-cell volumes were free from residual air. DSC runs at ordinary pressure (i.e., in standard aluminum pans) with mixtures of IMC and perfluorinated liquid were carried out to verify that the latter was inert.

Afterwards, the produced glass (CG) is pressurized to each pressure value, in the range from 0.1 to 300 MPa. After a stabilization time, a heating ramp at 2 K/min was initiated and the sample was heated from 300 K to 400 K in order to record the devitrification signal. Subsequently, the supercooled liquid is cooled back to ambient temperature at the same pressure and at around 2 K/min, obtaining a new conventional glass. Alternatively, the supercooled liquid was depressurized and, afterwards, cooled down to ambient temperature to produce the glass. As indicated in the main text, there is no appreciable difference in the onset of devitrification of the conventional glass produced by either of the described procedures.

In the case of the UG, each measured sample was kept at ambient temperature while the pressure was set to the working value, in the range from 0.1 to 300 MPa. After a stabilization time, a heating ramp at 2 K/min was initiated and the sample was heated from 300 K to 400 K. Each measurement was performed on a new sample. After the first calorimetric scan the glass transforms into the supercooled liquid, which is subsequently cooled at around 2 K/min and submitted to a second heating scan at a similar pressure to make certain that the now conventional glass falls on the same curve obtained for the conventional glass samples prepared by melting IMC.

## Additional Information

**How to cite this article**: Rodríguez-Tinoco, C. *et al*. Ultrastable glasses portray similar behaviour to ordinary glasses at high pressure. *Sci. Rep.* **6**, 34296; doi: 10.1038/srep34296 (2016).

## Supplementary Material

Supplementary Information

## Figures and Tables

**Table 1:** Experimental data and parameters used to test the validity of the Davies-Jones relation, equation (2).

Parameter	UG	CG
T_on_ [K] at P = 0.1 MPa	332	311
T_f_’ [K] at P = 0.1 MPa	282	312
∆α [1/K] (ref. 16)	4.30·10^−4^	4.36·10^−4^
∆C_P_ [J/molK]	150.74 (at 332 K) 167.37 (at 282 K)	138.23
v_m_ [cm^3^/mol] (ref. 16)	269.62	273.12
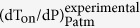 [K/GPa]	201	280, 254 (ref. 19)
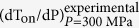 [K/GPa]	133	148
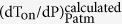 [K/GPa]	255	271
