# Supplementary material for: Ultrastable glasses portray similar behaviour to ordinary glasses at high pressure
Source: Sci Rep. 2016 Oct 3;6:34296. doi: 10.1038/srep34296 (PMC5046104; doi:10.1038/srep34296)
Supplement: Supplementary Information [file srep34296-s1.doc]

**SUPPLEMENTARY INFORMATION TO**

**Ultrastable glasses portray similar behaviour to ordinary glasses at high pressure**

C. Rodríguez-Tinoco^1^, M. González-Silveira^1^, M. Barrio^2^, P. Lloveras^2^, J. Ll. Tamarit^2,*^,

J. -L. Garden^3,4^, J. Rodríguez-Viejo^1,*^

^1^Grup de Nanomaterials i Microsistemes, Departament de Física, UniversitatAutònoma de Barcelona, 08193 Bellaterra

^2^Grup de Caracterització de Materials, ETSEIB, Departament de Física, UniversitatPolitècnica de Catalunya, Diagonal 647, 08028 Barcelona, Spain.

^3^CNRS, Inst NEEL, F-38000 Grenoble, France

^4^Univ. Grenoble Alpes, Inst NEEL, F-38000 Grenoble, France

**CALCULATION OF T_on_ vs PRESSURE FROM RELAXATION TIME**

In a previous work^1^, we inferred the relaxation time of glasses with different stability at ambient pressure from calorimetric data performed on these glasses at different heating rates.

According to that work, the relaxation time of these glasses, as well as the relaxation time of the equilibrium liquid, can be described by the expression developed by Casalini et al.^2^,


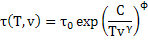


where
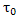
 and
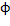
 are constants and
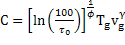
, with
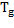
 the conventional value of glass transition temperature for IMC, 315 K, and
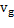
 the specific volume of a conventional glass at that temperature.
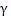
is the scaling factor. The particular values of these parameters were found by fitting the experimental data shown in Figure 1 from ref ^1^. In the case of the glass, the best fit is obtained with
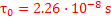
,
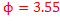
, and
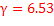
. In the case of the supercooled liquid,
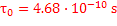
,
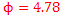
, and
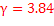
.

In ref. 1 all the experimental data was obtained at ambient pressure and, therefore, the employed expression for the density,
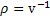
, was


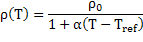


where
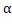
 is the system-dependent isobaric thermal expansion coefficient and
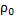
 is the density of the system at the reference temperature,
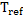
.

In the present work, however, we extend this model to measurements performed at variable pressure. As explained in the main text, we introduce the dependence of density on pressure through a pressure-dependent isothermal bulk modulus. The density of the system as a function of temperature and pressure is, therefore,


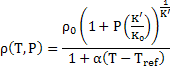


where
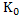
 is the bulk modulus of the system at ambient pressure and
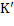
 is related to the dependence of the bulk modulus on pressure.

The bulk modulus of each glass at ambient pressure can be calculated using the known expression^3^,


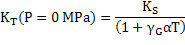


Where
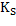
 is the adiabatic bulk modulus,
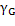
 is the Grüneisen parameter that we take approximately equal to the scaling factor and
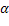
 is the isobaric thermal expansion coefficient. The adiabatic bulk modulus,
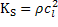
 was calculated by Ediger and co-workers from longitudinal sound velocity data on conventional and ultrastable IMC glasses^4^.
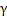
 and
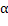
 are taken from ref. 1. From these data, we obtain, as indicated in the main text,
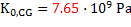
 and
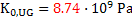
 for the bulk modulus of CG and UG at atmospheric pressure, respectively.

**SUPPLEMENTARY REFERENCES**

1. Rodríguez-Tinoco, C., Ràfols-Ribé, J., González-Silveira, M. & Rodríguez-Viejo, J. Relaxation dynamics of glasses along a wide stability and temperature range. *arXiv* 26 (2016). at <http://arxiv.org/abs/1603.08829>

2. Casalini, R., Mohanty, U. & Roland, C. M. Thermodynamic interpretation of the scaling of the dynamics of supercooled liquids. *J. Chem. Phys.* **125,** 014505 (2006).

3. Anderson, D. L. *Theory of the Earth*. (Blackwell Scientific Publications, 1989).

4. Kearns, K. L., Still, T., Fytas, G. & Ediger, M. D. High-modulus organic glasses prepared by physical vapor deposition. *Adv. Mater.* **22,** 39–42 (2010).
